# 3D Printing for Dental Applications

**DOI:** 10.3390/ma16144972

**Published:** 2023-07-12

**Authors:** Célio Gabriel Figueiredo-Pina, Ana Paula Serro

**Affiliations:** 1 Centro de Desenvolvimento de Produto e Transferência de Tecnologia (CDP2T), Escola Superior de Tecnologia de Setúbal, Instituto Politécnico de Setúbal, 2914-508 Setúbal, Portugal; celio.pina@estsetubal.ips.pt; 2 Egas Moniz Center for Interdisciplinary Research (CiiEM), Egas Moniz School of Health & Science, 2829-511 Almada, Portugal; 3 Centro de Física e Engenharia de Materiais Avançados (CeFEMA), Instituto Superior Técnico, Universidade de Lisboa, 1049-001 Lisboa, Portugal; 4 Centro de Química Estrutural (CQE), Institute of Molecular Sciences, Departamento de Engenharia Química, Instituto Superior Técnico, Universidade de Lisboa, 1049-001 Lisboa, Portugal

Due to increased life expectancy and greater concern among populations regarding oral health problems and aesthetics, in the last few years, there has been a growing demand for dental structures and devices to replace/restore missing/damaged teeth. Traditional production methods are mainly based on handcrafted fabrication techniques that are time-consuming, require great amounts of labor, and involve the use of several production support materials. However, recently, these production methods have started to be overtaken by digital technologies such as 3D printing, which has allowed for high levels of customization and the adoption of digital workflows that make practices more efficient and improve patient treatment outcomes. The introduction of these new cutting-edge technologies in the production system marks a clear revolution in dentistry, following the current trend towards Industry 4.0, which is characterized, among other aspects, by automation. The various 3D printing techniques may be grouped into vat polymerization, material jetting, powder–base fusion, sheet lamination, and direct energy deposition, and they allow for all kinds of materials to be processed, from synthetic materials to live tissues and organs. In dentistry, 3D printing can be used in the production of a wide range of devices, e.g., implants, inlays, onlays, overlays, surgical guides, custom models, aligners, surgical instruments, crowns, and prosthesis. Overall, the application of 3D printing technology in this field presents numerous economic, environmental, and social benefits, namely in terms of cost efficiency, as this form of technology involves lower energy consumption, material waste, and production times than conventional methods. It allows for decentralized manufacturing, thus enhancing the mass production of customized dental products at accessible costs, making them more accessible to the most disadvantaged sectors of society, which, on a global scale, has a positive impact on oral health. However, there are several challenges that must be overcome, for example, at the level of the completion of the pieces, their dimensional accuracy, and material production defects. The continued work of the scientific community in this area will help to fully establish 3D printing in the field of dentistry.

This Special Issue presents some of the most recent advances in this area; it brings together eleven articles (of which, two are reviews) that illustrate the high standard of research carried out in recent times by teams from twelve different countries and their efforts to develop new materials and processing methods based on additive manufacturing for dental applications.

In the first paper, Jung et al. [1] describe the manufacturing of dental crowns made of 5 mol% yttria partially stabilized zirconia through the use of digital light processing (DLP). They optimized the properties of the printing material (e.g., the particle size and solid and dispersant contents in the suspensions), as well as the processing parameters, to achieve adequate mechanical properties (flexural strength and Weibull modulus) and optical translucency. To ensure high dimensional accuracy, the initial dimensions of the crowns were precisely determined by considering the increase and decrease in the dimensions during photopolymerization and sintering, respectively. The photopolymerization time was adjusted to guarantee good bonding between the layers. Debinding was performed through a multi-step process with a slow heating rate to avoid the formation of defects. The sintered crowns presented high relative densities, high cubic phase content, and no visible defects within or between the layers. 

Al-Dulaijan et al. [2] investigated the effect of printing orientation (0, 45, and 90 degrees) and post-curing time (30, 60, 90, and 120 min) on the surface roughness (average roughness, Ra) and hardness (Vickers hardness, VH) of two 3D-printed commercial denture base resins after thermocycling (10,000 cycles) and compared them with a conventional heat-polymerized (HP) resin. The printing orientation and post-curing time did not affect the Ra of the printed resins. Moreover, in general, the printed resins did not show any significant changes in their Ra when compared with the HP resins, although some exceptions were observed for 45-degree orientation and high post-curing times (90 and 120 mins). Concerning hardness, the printed resins showed lower VH values than the HP resin. The VH of the printed resins improved with the post-curing time but was affected differently by the printing orientations, depending on the type of resin.

In another study, Nimmawitt et al. [3] studied the stress distribution on the bone tissue and bone–implant interface of a customized anatomic root–analog dental implant (RAI) by means of finite element analysis (FEA) for different bone densities. They measured the Von Mises stress in the bone tissues and bone–implant interfaces. To validate the computer-aided designed (CAD) models, the RAI was 3D printed through a laser powder–bed fusion (L-PBF) approach. The results showed that none of the RAI designs led to plastic deformation or fracture, giving rise to lower stress than the ultimate tensile stress of natural bone and the implant. RAIs have been demonstrated to possess a more suitable stress distribution pattern around the bone tissue and the bone-implant interface than conventional screw-type implants. The porous structure reduced the stress in the cancellous bone area with a very thin cortex and low-density trabeculae.

Jiang et al. [4] proposed an innovative form of 3D printing technology with a submersion-light apparatus for dental applications that increases the suspension capacity of the powder in a ceramic slurry. A double-sided constraint method for the cured layer combined the advantages of the top-down and bottom-up photopolymerization methods. They prepared a zirconia-based slurry containing 6-Hexanediol diacrylate (HDDA) and a photo-initiator and submerged a light engine box in the slurry which emitted a sliced layer pattern to induce photopolymerization. The obtained green body was sintered in a two-step process, and the shrinkage ratios in all dimensions were determined. Moreover, the HV, density, and flexural strength of the sintered piece were also characterized. They produced a three-unit zirconia dental bridge and proved that it was possible to obtain a clinically acceptable marginal gap.

The 3D printing accuracy of partial-arch models according to outer wall thickness was evaluated in an in vitro study by Shin et al. [5]. Anterior and posterior partial-arch models with different outer wall thicknesses were designed and printed by means of stereolithography (SLA), and a trueness analysis was performed to analyze the effect of wall thickness and printing direction accuracy. The trueness values in the anterior and posterior partial-arch groups indicated that the accuracy increased with the outer wall thickness and that the fully filled model was the most accurate. The anterior partial-arch group had lower printing accuracy than the posterior partial-arch group, which appears to be due to the volume differences varying with the size of the model. Furthermore, the trueness of the partial-arch model was better than that of the full-arch model, and models with thick outer walls at 60 degrees were highly accurate.

Pinho et al. [6] performed tensile and stress relaxation tests to evaluate the influence of build orientation and geometry on the mechanical properties of 3D-printed poly(ε-caprolactone) pieces for dental use. Due to their intended application, the tests were repeated after aging in artificial saliva. Specimens with parallelepiped and tubular geometry were printed with longitudinal and transversal toolpaths. The obtained results allowed us to conclude that geometry critically influences mechanical properties; the best mechanical properties were obtained for parallelepiped geometry with a longitudinal impression. Moreover, the process of aging in artificial saliva negatively influenced both of the mechanical properties considered in the study.

In another study, Ziebowicz et al. [7] produced pieces of a cobalt-based alloy through direct metal laser sintering (DMLS) and deposited a zirconium oxide layer of 50 nm over them using the atomic layer deposition (ALD) method. The coating improved the material’s physicochemical properties for prosthetic restorations, leading to roughness reduction, turning the surface more hydrophobic, and increasing the corrosion resistance. Moreover, it limited the bacterial adhesion to the substrate in comparison with the bare alloy. This may be of added value in dealing with prosthetic stomatopathy, which is a common complex problem in populations who use removable dentures, since it can reduce the improper performance of the devices and allergic reactions, as well as the multiplication of bacteria on their surface.

The main goal of the study by Sabbah et al. [8] was to assess the effect of printing layer thickness on repeatability and surface roughness and determine the effect of layer thickness and storage time on the dimensional stability of 3D-printed dies. Molar crowns were printed by means of stereolithography (STL). Repeatability was evaluated by linear and area measurements. Dimensional stability was analyzed as a function of storage time. The surface roughness parameters were also determined. Although high repeatability and comparable surface roughness were found in all cases, differences were observed in the linear dimensions and surface areas. After 3 weeks of storage, dimensional changes were observed. The results allowed the researchers to conclude that changes in the printing layer thickness do not affect the repeatability or the surface roughness of the product. However, changes in the layer thickness and storage time influence the dimensional stability of the printed dies.

The final experimental study to be included in this Special Issue [9] compared the bone healing and implant stability of three types of titanium dental implants (a threaded implant, a 3D-printed implant without spikes, and another implant with spikes) through in vivo tests on beagles. The mandibular premolars and first molars of the animals were removed, and after twelve weeks, the three types of implants were randomly inserted into the edentulous ridges of the dogs. Then, every two weeks, stability measurements and radiographic analyses were performed until twelve weeks. At that time, the animals were sacrificed, and the bone-to-implant contact and bone area fraction occupied were compared. Although at the time of implantation, the implant stability was lower for the 3D-printed implant with spikes than for the threaded implant, after twelve weeks, no significant differences were observed. Concerning the 3D-printed implants without spikes, results comparable to the threaded implants were found for the stability measurements, bone-to-implant contact, and bone area fraction occupied. Finally, histomorphometrical analysis led to similar results for the three implants. 

The last two articles included in this Special Issue are reviews of the most recent advances in the 3D printing of two materials relevant to the dental field: a ceramic (zirconia) and a polymer (polyetheretherketone, PEEK), respectively. The first [10] presents the actual state of the art of the additive manufacturing of zirconia-based materials for dental applications and makes a comparative analysis of the properties of the obtained materials. Stereolithography (SLA) and digital light processing (DLP) were the techniques most focused upon in the analyzed studies and those that led to the most promising results. Other techniques, such as robocasting (RC) and material jetting (MJ), were also explored, with good results being obtained. The authors concluded that despite the great advances in the area and the disruptive technological progress that they represent, some concerns still remain regarding the dimensional accuracy, resolution, and mechanical strength of the pieces. The second review [11] was centered on the mechanical properties of fused deposition modeling (FDM) 3D-printed PEEK and its relevance in dental restoration. It aimed to identify the optimal printing parameters to produce pieces with suitable properties. The authors make reference to the fact that the studies were difficult to compare due to the variability of the printing parameters and the types of PEEK; despite this, they highlight the advantage of using high infill rates, high chamber temperatures (close to the printing temperature), and heat post-treatments to obtain 3D PEEK pieces with adequate properties for dental restorations. 

Overall, the works presented in this Special Issue show that a considerable amount of research is being conducted in order to turn 3D printing technologies into a reality in the dentistry field, underlying its importance in improving patients’ health and quality of life and reducing the economic impact of oral treatments. The editors of this Special Issue expect that its readers will find the works presented herein to be interesting and stimulating and that they may contribute to generating new ideas for their future research.
